# Using a penalized likelihood to detect mortality deceleration

**DOI:** 10.1371/journal.pone.0294428

**Published:** 2023-11-16

**Authors:** Silvio C. Patricio, Trifon I. Missov

**Affiliations:** Interdisciplinary Centre on Population Dynamics, University of Southern Denmark, Odense, Denmark; Cairo University, EGYPT

## Abstract

We suggest a novel method for detecting mortality deceleration by adding a penalty to the log-likelihood function in a gamma-Gompertz setting. This is an alternative to traditional likelihood inference and hypothesis testing. The main advantage of the proposed method is that it does not involve using a *p*-value, hypothesis testing, and asymptotic distributions. We evaluate the performance of our approach by comparing it with traditional likelihood inference on both simulated and real mortality data. Results have shown that our method is more accurate in detecting mortality deceleration and provides more reliable estimates of the underlying parameters. The proposed method is a significant contribution to the literature as it offers a powerful tool for analyzing mortality patterns.

## 1 Introduction

Human death-rate patterns are astoundingly log-linear over a wide range of adult ages. The Gompertz distribution [1] with an exponentially increasing hazard function captures this accurately. The theory of unobserved heterogeneity and the associated frailty model [2] predicts a downward deviation at the oldest ages, to which only the most robust individuals in the population survive. Detecting such a deceleration in real data is not always successful [3, 4], even though the vast majority of studies indicate that death rates at older ages increase at lower rates and can even level off [5–15]. In a frailty model setting, testing for mortality deceleration is equivalent to testing whether the non-negative frailty parameter is strictly positive.

Formally, denote by *X* a non-negative continuous random variable that describes individual human lifespans (complete or after a given adult age). If *X* has a Gompertz distribution with parameters *a* and *b*, where *a* is the mortality level at the initial age and *b* is the rate of aging, the associated hazard function (force of mortality) at time *x*

$$\begin{array}{c} \mu \left(x\right)=\underset{\varepsilon ↓0}{\text{lim}\;}P\left(x\leq X<x+\varepsilon |X\geq x\right), \end{array}$$

is *μ*(*x*) = *ae*^*bx*^. The Gompertz hazard [1] captures adequately the log-linear acceleration observed in death rates in the adult age range [16, 17]. The use of the Gompertz model is also justified by extreme value and evolutionary theories [18, 19]. It is, therefore, appropriate to assume that the general schedule for the individual risk of dying follows a Gompertz law.

Deviations from the log-linear mortality pattern, especially at the oldest ages, can be attributed to the intrinsic diversity among individuals in the study population. While standard survival models capture the variability due to measurable risk factors, it is their extension, the frailty models, that also incorporate the effect of unobserved heterogeneity. The latter can reflect, among others, genetic predispositions to certain diseases [20], mental health, or the general quality of life. The unobserved heterogeneity among individuals affects their endowment for longevity [21, 22] and, to take this into account, standard frailty models introduce a random effect called *frailty*. This random effect, considered in general to be non-negative, is incorporated into the individual hazard as a multiplicative factor that reflects one’s unobserved susceptibility to death [2].

The force of mortality for an individual with frailty *Z* = *z* is

$$\begin{array}{c} \mu (x\;|\;Z=z)=z\;\mu (x)\;. \end{array}$$

Frail individuals have high values of *z* and, therefore, tend to die first. The connection between frailty and baseline mortality, operating in a multiplicative manner, is justified by the observed death-rate patterns of elderly humans [10, 12, 18, 23].

The estimation of frailty models requires specifying a distribution for the random and unobserved frailty *Z* and studying the resulting marginal distribution [2, 18]. The main property of frailty’s distribution is the regular variation at zero of its density [18, 23]. Distributions that meet this condition are the gamma, beta, truncated normal, log-logistic, and even Weibull distribution [23].

Among all admissible distributions, we opt for the gamma distribution as it has a well-defined mathematical structure that facilitates both analytical and numerical computations, and it is also flexible and capable of capturing a wide range of shapes for the frailty component [2, 18]. For a gamma-distributed frailty *Z* with E(Z)=1 and $VAR\left(Z\right)=\sigma^{2}$, the force of mortality of the population, i.e., the marginal hazard is

$$\begin{array}{c} \bar{\mu }\left(x\right)=\frac{ae^{bx}}{1+\sigma^{2}\frac{a}{b}(e^{bx}-1)} \end{array}$$ (1)

(see [2, 24] for all technicalities). Note that the variance of *Z* is often denoted by *γ* because it is also equal to the squared coefficient of variation of the distribution of frailty among survivors to any age *x*. If *σ*^2^ > 0, the force of mortality for the population $\bar{\mu }\left(x\right)$ starts deviating from the exponential pattern with increasing *x* and reaches an asymptote *b*/*σ*^2^. When *σ*^2^ = 0, i.e., when there is no unobserved heterogeneity, the model for the population reduces to the (Gompertz) model for individuals with an exponentially increasing hazard function *μ*(*x*) = *ae*^*bx*^.

Testing for mortality deceleration in this setting reduces to statistical testing whether *σ*^2^ = 0 given the alternative *σ*^2^ > 0. The frailty parameter *σ*^2^ can take a value on the boundary of the parameter space (*σ*^2^ = 0). This violates the standard underlying assumptions about the asymptotic properties of likelihood-based inference and statistical hypothesis testing [25]. As a result, the asymptotic distribution of the maximum likelihood estimator may not be Gaussian.

In this paper, we treat the problem of identifying whether *σ*^2^ > 0 or *σ*^2^ = 0 as a model misspecification problem, i.e., we consider the gamma-Gompertz model when it is the Gompertz model that actually holds. In this setting, we suggest adding a penalty from the log-likelihood function. This penalty will be responsible for shrinking *σ*^2^ to zero when there is no heterogeneity, as well as for adding a small bias to the Maximum Likelihood Estimator (MLE) when the effect of unobserved heterogeneity is non-negligible. We carry out Monte Carlo simulation experiments to evaluate the accuracy and precision of the estimates obtained by maximizing the likelihood function, on the one hand, and the penalized likelihood function, on the other.

In Section 2, we formulate the model misspecification problem and introduce inference methodology taking advantage of the maximum a posteriori probability (MAP). Then we carry out a Monte Carlo simulation study to compare the performance of maximizing a standard and a penalized likelihood. In Section 3, we compare the latter on mortality data for France, Japan and the USA. Section 4 discusses the advantages and drawbacks of applying our method to detect heterogeneity (deceleration) in mortality patterns.

## 2 Methodology

Suppose X is a random sample with a cumulative distribution function *G*(*x*), and we fit the incorrect family of densities {f(x;θ),θ∈Θ} to the data using MLE. The misspecified log-likelihood is

$$\begin{array}{c} \ell \left(\theta ;X\right)=\sum_{i=1}^{n}\;\text{log}\;f\left(X_{i};\theta \right). \end{array}$$

Applying the law of large numbers, we get in the limit what the misspecified log-likelihood function ℓ(θ;X) looks like for each θ∈Θ (see the right-hand side below):

$$\begin{array}{c} \frac{1}{n}\ell \left(\theta ;X\right)=\frac{1}{n}\sum_{i=1}^{n}\;\text{log}\;f\left(X_{i};\theta \right)\overset{a.s}{\to }E_{g}(\;\text{log}\;f(X_{1};\theta ))=\int_{\text{Im}(X_{1})}\;\text{log}\;f\left(x;\theta \right)dG\left(x\right)\;. \end{array}$$ (2)

Assume there is no heterogeneity in the data (*σ*^2^ = 0), and we fit a gamma-Gompertz model. In other words, we observe an exponential death-rate increase in the data, but we estimate a model that implies a downward deviation from the exponential at the oldest ages. As shown in Eq 2, we will estimate *σ*^2^ close to but never equal to zero.

In this model setting, the standard technique is to estimate both the Gompertz and the gamma-Gompertz models and compare their goodness of fit. However, minor changes in the data can result in different models being selected, which can reduce prediction accuracy and lead to misinterpretations about the mortality deceleration and the mortality plateau. [25] derive the asymptotic distribution of the likelihood ratio test statistic to detect heterogeneity. Here, we suggest an alternative that does not involve hypothesis testing. Using the latter has been widely discussed and rethought in the Statistics community [26–29], especially concerning the arbitrary choice of the *α*-level (most often 0.1, 0.05, or 0.01) and sample size issues.

Maximum likelihood estimators, obtained by maximizing the log-likelihood function, often have low bias and large variance. Estimation accuracy can sometimes be improved by shrinking some parameters to zero [30]. The associated shrinkage estimator improves the overall accuracy, promotes parsimony and makes the parameter estimates more stable by reducing their sensitivity to minor changes in the data, at the expense of introducing a small bias to reduce the variance of the parameters. This class of estimators is implicit in Bayesian inference and penalized likelihood inference. Using shrinkage estimators is applied as an alternative to hypothesis testing. Lasso, Ridge, and Stein-type estimators are the most widely used examples of penalizing methods [31].

### 2.1 Inference

Let *D*_*x*_ be the number of deaths in a given age interval [*x*, *x*+ 1) for *x* = 0, …, *m*, and *E*_*x*_ denote the number of person-years lived in the same interval [32, 33]. Define $D={\left(D_{0},D_{1},\ldots ,D_{m}\right)}^{⊤}$ and $E={\left(E_{0},E_{1},\ldots ,E_{m}\right)}^{⊤}$. In addition, let **θ** = (*a*, *b*, *σ*^2^)^⊤^ ∈ *Θ* be the parameter vector that characterizes the force of mortality at age *x* of the gamma-Gompertz model given by Eq 1. Finally, we assume that the number of deaths and the number of person-years exposed to the risk of dying can be observed.

Assume *D*_*x*_ are Poisson-distributed with $E\left(D_{x}\right)=VAR\left(D_{x}\right)=\mu \left(x;\theta \right)E_{x}$ for *x* = 0, …, *m* [32]. Under this assumption, the log-likelihood function for **θ** = (*a*, *b*, *σ*^2^)^⊤^ is given by

$$\begin{array}{c} \ell \left(\theta \right)=\ell \left(\theta |D,E\right)=\sum_{x=0}^{m}[D_{x}\;\text{ln}\;\mu \left(x;\theta \right))-E_{x}\;\mu \left(x;\theta \right)]. \end{array}$$ (3)

Maximizing ℓ(θ) with respect to **θ** = (*a*, *b*, *σ*^2^)^⊤^ yields the maximum-likelihood (ML) estimate $\hat{\theta }$. Suppose the data come from a Gompertz distribution, and we estimate a gamma-Gompertz model, i.e., the true value of *σ*^2^ is 0. Then, for each set of fixed model parameters θ∈Θ, we can use the limit on the right-hand side of (2) to calculate the expected log-likelihood as a function of *σ*^2^.

**Fig 1:**
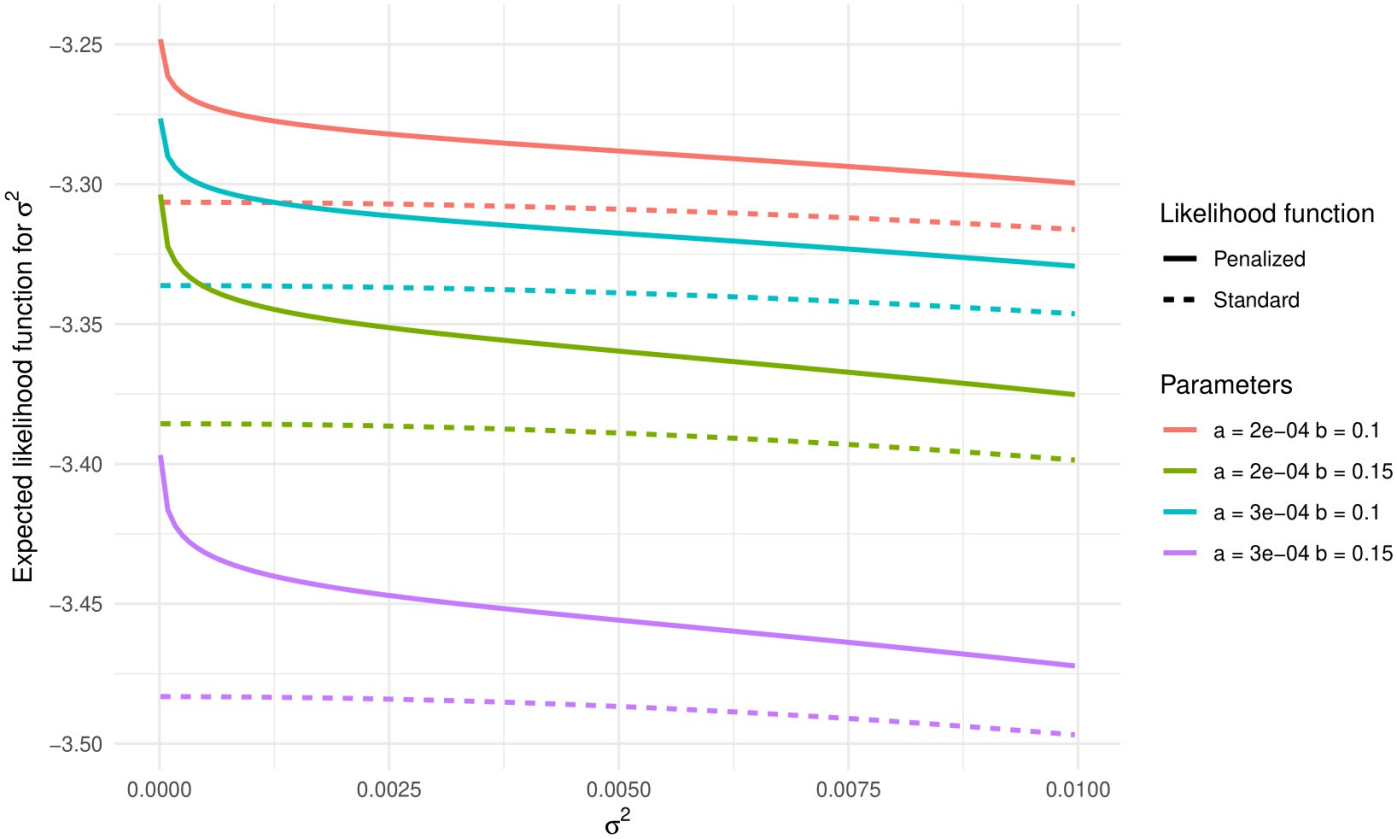
Expected log-likelihood function for *σ*^2^ according to the right-hand side of (2) when the data are generated by the Gompertz distribution (*σ*^2^ = 0), and the fitted model is the gamma-Gompertz.

Fig 1 shows the expected log-likelihood function for four pairs of fixed values for *a* and *b*. We can see that the likelihood (dashed line) might not be “concave enough,” especially when the true value of *σ*^2^ is close to 0, to allow direct optimization. Indeed, the function is almost flat when *σ*^2^ ≤ 0.005, which means that these values are (almost) equally likely. In such cases, using a penalty function, also known as a *regularization term* or a *prior distribution*, can be instrumental in increasing concavity at the expense of introducing some constraints or biases into the estimation process [31, 34].

**Fig 2:**
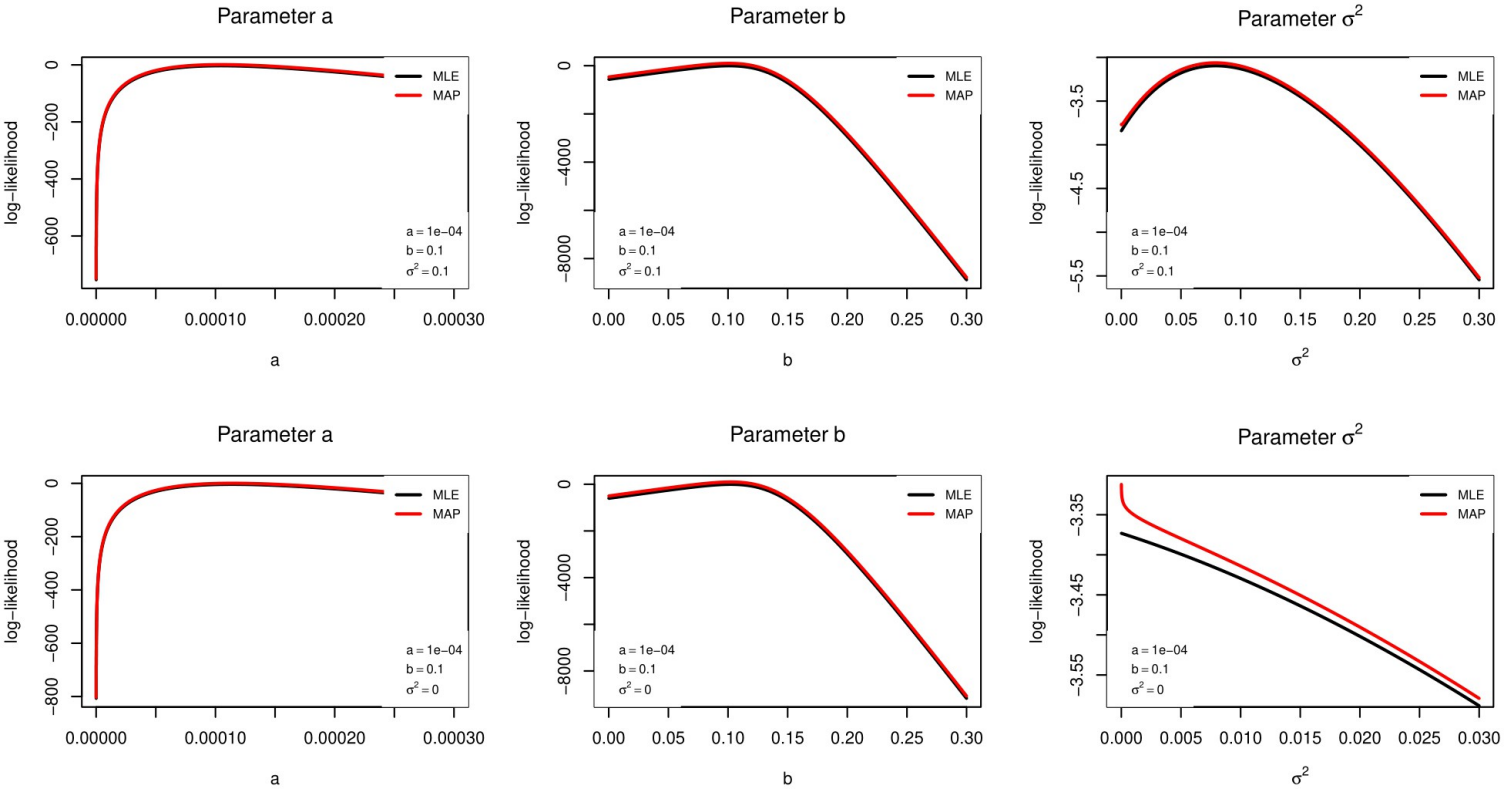
Plots of the profile log-likelihood (first row) and penalized log-likelihood functions (second row) of the parameters. In the first row, we used synthetic data from a gamma-Gompertz model with parameters *a* = 0.0001, *b* = 0.1 and *σ*^2^ = 0.1; in the second row, we from a Gompertz model with parameters *a* = 0.0001 and *b* = 0.1.

Let us now define a penalized log-likelihood function as

$$\begin{array}{c} \ell_{p}\left(\theta \right)=\ell \left(\theta \right)+p\left(\sigma^{2}\right)\;, \end{array}$$ (4)

where ℓ(θ) is the standard log-likelihood (Eq 3), while *p*(*σ*^2^) is a penalty function. The penalized maximum-likelihood estimate is obtained by maximizing $\ell_{p}\left(\theta \right)$ with respect to **θ** = (*a*, *b*, *σ*^2^)^⊤^. If the effect of unobserved heterogeneity is negligible, i.e., when there is no mortality deceleration, we aim to estimate *σ*^2^ equal to 0. For that, the penalty *p*(*σ*^2^) must be a non-increasing monotonic continuous function and $\underset{\sigma^{2}↓0}{\lim}\;p(\sigma^{2})>p(\sigma^{2})$ for all *σ*^2^ > 0, i.e, the penalty function reaches its maximum for *σ*^2^ = 0. The last condition ensures that when there is no unobserved heterogeneity, maximizing (4) yields a frailty parameter exactly equal to 0. An example is shown in Figs 1 and 2.

In a Bayesian framework, maximizing (4) is equivalent to maximizing a posterior distribution in a setting in which $e^{p(\sigma^{2})}/C_{p}$, $C_{p}:=\int e^{p(\sigma^{2})}d\sigma^{2}<\infty$, is taken as a prior distribution of *σ*^2^. This procedure yields the maximum a posteriori probability (MAP) estimator, widely used in image and video processing [35–37].

Given that *σ*^2^ characterizes the variance of frailty, it is standard to assign an inverse gamma prior distribution to it [38]. The inverse gamma distribution, known for its heavy-tail, effectively maintains a greater probability mass away from zero than the gamma distribution. Note that the mode of the inverse gamma distribution is consistently positive, whereas the mode of the gamma distribution can potentially be zero [39]. As we aim to test whether *σ*^2^ = 0 or *σ*^2^ > 0, we will use the log-kernel of the gamma distribution to define the penalty function as

$$\begin{array}{c} p\left(\sigma^{2}\right)=-\lambda (\sigma^{2}+\;\text{ln}\;\sigma^{2}) \end{array}$$ (5)

for some non-negative λ. When λ < 1, using (5) is equivalent to specifying a gamma prior distribution for *σ*^2^ with parameters *α* = 1 − λ and *β* = λ (maximized at *σ*^2^ = 0). When *m* → ∞, the effect of the penalty diminishes regardless of the size of λ. For human life table data *m* is finite.

The penalty parameter λ ≥ 0 is a constant that controls the relative impact of the penalty function on the estimates. When λ = 0, the penalty term has no effect, and maximizing the penalized likelihood will produce the standard maximum likelihood estimator (MLE). However, as λ → ∞, the impact of the penalty grows, and the maximum penalized likelihood estimates for *σ*^2^ will approach zero, providing high precision, but low accuracy.

Choosing λ is sensible in a wide range of applications [40, 41]. Therefore, we carry out a pilot simulation study, in which we find that choosing $\lambda =\frac{1}{2}$ provides similar precision to the one by MLE when *σ*^2^ > 0, but better accuracy and precision when *σ*^2^ = 0 (simulation results are presented in the next subsection). As a result, the final expression for the penalized log-likelihood we propose is

$$\begin{array}{c} \ell_{p}\left(\theta \right)=\sum_{x=0}^{m}[D_{x}\;\text{ln}\;\mu \left(x;\theta \right)-E_{x}\;\mu \left(x;\theta \right)]-\frac{1}{2}(\;\text{ln}\;\sigma^{2}+\sigma^{2}). \end{array}$$ (6)

The expected penalized log-likelihood function for *σ*^2^ is shown in Fig 1 (solid line). As the penalty function is maximized at *σ*^2^ = 0 and the log-likelihood function is almost flat for *σ*^2^ < 0.005, the penalized log-likelihood function has a distinct maximum at *σ*^2^ = 0, reflecting that zero is the most likely value for *σ*^2^.

From a Bayesian perspective, choosing $\lambda =\frac{1}{2}$ provides an informative prior distribution for *σ*^2^. As for human populations, we are likely to estimate *σ*^2^ < 1 [42], the specified prior will provide for *σ*^2^ a distribution with a mode equal to zero, a median equal to 0.4549, and mean equal to 1. Furthermore, the prior provides a probability mass of 0.6826 in the interval (0, 1].

Fig 2 shows the log-likelihood and penalized log-likelihood functions for all parameters when *σ*^2^ > 0 (first row) and *σ*^2^ = 0 (second row). When *σ*^2^ > 0, the penalty function affects neither the shape of the log-likelihood nor the location of its maximum. However, when *σ*^2^ = 0, adding a penalty yields a higher maximum at 0. Moreover, when *σ*^2^ = 0, the first and second derivatives of the penalized log-likelihood are higher than their respective counterparts of the log-likelihood. As a result, derivative-based optimization methods may reach the maximum point faster, and the estimator $\hat{\sigma^{2}}$ may have a smaller variance.

### 2.2 Monte Carlo simulations

We carry out Monte Carlo simulations to explore the performance of the MAP and ML methods in estimating the gamma-Gompertz model parameters. We use the R software [43] to maximize the log-likelihood and the penalized log-likelihood functions via the optim function applying as a pre-step differential evolution [44, 45]. The performance of the ML and MAP estimators are evaluated by calculating two measures: the bias and the standard deviation.

We generate 10,000 random samples from this model for some parameter values (scenarios with sample sizes of 2,000 and 5,000 were also considered, and are presented in the S1 Appendix). From these samples, we generate life tables and use them to estimate model parameters via the MAP and MLE methods. This process was repeated 2,000 times. In the presence of unobserved heterogeneity, the true parameter values are *a*_1_ = 0.0001 and *a*_2_ = 0.00001 for *a*, *b*_1_ = 0.1 and *b*_2_ = 0.15 for *b*, and $\sigma_{1}^{2}=0.2$ and $\sigma_{2}^{2}=0.8$ for *σ*^2^. When there is no heterogeneity (*σ*^2^ = 0), the true parameter values are *a*_1_ = 0.0001, *a*_2_ = 0.0003 and *a*_3_ = 0.0005 for *a*, and *b*_1_ = 0.09, *b*_2_ = 0.10 and *b*_3_ = 0.11 for *b*.

The simulation results are presented in Table 1. In the presence of unobserved heterogeneity, both methods underestimate *b* and *σ*^2^. They also introduce a small positive bias to *a*, the one provided by ML estimator being slightly smaller. However, in general the ML and MAP estimators perform equally well, with a similar bias and standard deviation.

**Table 1:** Simulation results: Gamma-Gompertz model and sample size 10,000.

	There is heterogeneity											
	MLE estimator						MAP estimator					
	Bias			Standard deviation			Bias			Standard deviation		
Parameter	*a*	*b*	*σ* ^2^	*a*	*b*	*σ* ^2^	*a*	*b*	*σ* ^2^	*a*	*b*	*σ* ^2^
$\left(a_{1},b_{1},\sigma_{1}^{2}\right)$	0.000053	-0.000051	-0.000223	0.000051	0.001499	0.020721	0.000055	-0.000134	-0.001626	0.000052	0.001502	0.020791
$\left(a_{1},b_{1},\sigma_{2}^{2}\right)$	0.000060	-0.000292	-0.007787	0.000056	0.001784	0.035822	0.000061	-0.000354	-0.009229	0.000056	0.001783	0.035795
$\left(a_{1},b_{2},\sigma_{1}^{2}\right)$	0.000077	0.000131	0.004431	0.000056	0.002181	0.020569	0.000080	0.000015	0.003096	0.000057	0.002186	0.020631
$\left(a_{1},b_{2},\sigma_{2}^{2}\right)$	0.000085	-0.000262	-0.003547	0.000061	0.002557	0.034714	0.000087	-0.000348	-0.004920	0.000061	0.002556	0.034687
$\left(a_{2},b_{1},\sigma_{1}^{2}\right)$	0.000007	-0.000349	-0.003349	0.000008	0.001315	0.019464	0.000008	-0.000417	-0.004597	0.000008	0.001318	0.019523
$\left(a_{2},b_{1},\sigma_{2}^{2}\right)$	0.000009	-0.000635	-0.013592	0.000008	0.001515	0.032551	0.000009	-0.000683	-0.014810	0.000008	0.001514	0.032528
$\left(a_{2},b_{2},\sigma_{1}^{2}\right)$	0.000009	-0.000170	0.002295	0.000008	0.001963	0.019377	0.000009	-0.000268	0.001078	0.000008	0.001966	0.019430
$\left(a_{2},b_{2},\sigma_{2}^{2}\right)$	0.000011	-0.000650	-0.007809	0.000009	0.002273	0.032534	0.000011	-0.000721	-0.009014	0.000009	0.002272	0.032510
	There is heterogeneity											
	MLE estimator						MAP estimator					
	Bias			Standard deviation			Bias			Standard deviation		
Parameter	*a*	*b*	*σ* ^2^	*a*	*b*	*σ* ^2^	*a*	*b*	*σ*^2^(10^−16^)	*a*	*b*	*σ*^2^(10^−15^)
(*a*_1_, *b*_1_, *σ*^2^)	0.000005	-0.000055	0.000181	0.000006	0.000875	0.008460	0.000007	-0.000239	0.125942	0.000006	0.000791	0.6308937
(*a*_1_, *b*_2_, *σ*^2^)	0.000006	-0.000050	0.000222	0.000006	0.000968	0.008907	0.000007	-0.000407	0.060433	0.000006	0.000870	0.1460452
(*a*_1_, *b*_3_, *σ*^2^)	0.000006	-0.000057	0.000428	0.000006	0.001082	0.009726	0.000008	-0.000305	0.089035	0.000006	0.000978	8.212895
(*a*_2_, *b*_1_, *σ*^2^)	0.000011	0.000138	0.001864	0.000016	0.000913	0.009010	0.000016	-0.000198	0.004470	0.000015	0.000811	0.294978
(*a*_2_, *b*_2_, *σ*^2^)	0.000014	0.000117	0.001094	0.000016	0.001042	0.009873	0.000019	-0.000264	0.003661	0.000015	0.000859	4.922260
(*a*_2_, *b*_3_, *σ*^2^)	0.000015	0.000164	0.002204	0.000016	0.001150	0.010369	0.000022	-0.000216	0.007167	0.000016	0.001017	3.551245
(*a*_3_, *b*_1_, *σ*^2^)	0.000018	0.000143	0.001062	0.000025	0.000979	0.009712	0.000027	-0.000138	0.001124	0.000025	0.000876	1.898573
(*a*_3_, *b*_2_, *σ*^2^)	0.000024	0.000076	0.000505	0.000025	0.001057	0.009721	0.000030	-0.000112	0.001177	0.000025	0.000942	8.114498
(*a*_3_, *b*_3_, *σ*^2^)	0.000025	0.000117	0.001427	0.000025	0.001178	0.009999	0.000033	-0.000171	0.001415	0.000024	0.000990	0.000025

In the absence of unobserved heterogeneity, the ML estimator provides again a smaller bias for *a* and *b* than the MAP estimator. However, in this case, the MAP method estimates more precisely the frailty parameter *σ*^2^, with a bias and a standard deviation close to zero (∝ 10^−15^). The MAP estimator also provides a slight reduction in the standard deviation of parameter *b*. Similar results were found for smaller samples, Tables.3 and 4 in S1 Appendix present the simulation results for sample sizes 2,000 and 5,000 respectively.

By the Monte Carlo simulation we also calculate the proportion of trials in which MAP estimates *σ*^2^ > 0 when the true values is *σ*^2^ = 0 (error type I), as well as the proportion of trials in which MAP estimates *σ*^2^ = 0 when the true values is *σ*^2^ > 0 (error type II). Based on our simulations, the type I errro equals 0.001502, while the type II error is 0.001126.

The Monte Carlo simulations show that using a penalizing likelihood function (6) is an alternative to hypothesis testing, the latter being dependent on the asymptotic distribution of the ML estimator, sample size and the arbitrary choice of the *α*-level [25].

## 3 Performance of MAP and ML estimators on HMD data

In this section, we estimate the gamma-Gompertz model via ML and MAP using mortality data from the Human Mortality Database [46]. We take exposures and raw death counts for the female population of France, Japan and the USA in the years 1960, 1980, 2000, and 2020, after age 70. We apply again R [43] to compute the ML and MAP estimates of $\theta ={\left(a,b,\sigma^{2}\right)}^{\prime }$ by using differential evolution. We use the mean squared error given by

$$\begin{array}{c} MSE=\frac{1}{n}\sum_{x=0}^{m}{\left(\;\text{ln}\;m_{x}-\;\text{ln}\;\bar{\mu }\left(x;\hat{\theta }\right)\right)}^{2}, \end{array}$$

to assess the goodness of fit.

Table 2 shows the results of applying ML and MAP methods to the datasets described above. The MAP estimator provides lower MSEs in 8 of the 12 datasets. When the standard ML method estimates *σ*^2^ < 10^−4^, our novel method estimates *σ*^2^ = 0 and provides a smaller MSE. This suggests that the MAP provides a slightly better fit to the data. Overall, MAP performs better than ML when unobserved heterogeneity is not detected, and while for estimates of ${\hat{\sigma }}^{2}>0$ ML has a slight advantage.

**Table 2:** Life expectancy: gamma Gompertz–Makeham model and ML estimates.

Country	Year	ML Estimates				MAP Estimates			
		*a*	*b*	*σ* ^2^	MSE	*a*	*b*	*σ* ^2^	MSE
France	1960	0.003582	0.107599	0.016726	0.100588	0.003593	0.107483	0.015902	0.100540
	1980	0.002210	0.112032	0.003393	0.045776	0.002220	0.111729	0.003117	0.046747
	2000	0.001247	0.117749	0.000001	0.073648	0.001250	0.117689	0	0.073262
	2020	0.000957	0.119494	0.000002	0.094243	0.000960	0.119416	0	0.093697
Japan	1960	0.004782	0.105858	0.039989	0.011366	0.004782	0.105845	0.039593	0.011493
	1980	0.002009	0.117886	0.015251	0.053944	0.002009	0.117941	0.015942	0.053328
	2000	0.001140	0.115268	0.000015	0.064604	0.001142	0.115118	0	0.063728
	2020	0.000575	0.125814	0.000233	0.104944	0.000574	0.125870	0.000225	0.105597
USA	1960	0.004701	0.095797	0.032146	0.111711	0.004699	0.095814	0.032802	0.110740
	1980	0.003612	0.093688	0.000001	0.054483	0.003609	0.093720	0	0.054652
	2000	0.002712	0.100566	0.000003	0.030967	0.002714	0.100540	0	0.030873
	2020	0.002473	0.101652	0.000001	0.022735	0.002476	0.101612	0	0.022618

The results from the real-data application back up the results from the Monte Carlo simulations in Section 2. In the presence of unobserved heterogeneity, the MLE method provides the most precise and accurate estimates. The MAP method, though, has just slightly lower precision. On the other hand, in the absence of unobserved heterogeneity, the MAP provides a smaller bias and variance in its estimates compared to MLE.

### 3.1 Examples when MAP and ML estimators yield different outcomes

Using MAP and ML estimators does not always lead to the same statistical inference. One of them can detect heterogeneity in cases when the other does not. We will illustrate this on HMD data for the Japanese female population in 2009 and the French female population born in 1848, ages 70+. To assess the goodness of fit, we will use MSE again.

For Japanese females in 2009, ML yields estimates ${\hat{\theta }}_{MLE}={\left(0.006359,0.133805,0.070513\right)}^{\prime }$ with standard errors *SE*(*a*) = 0.000188, *SE*(*b*) = 0.002263 and *SE*(*σ*^2^) = 0.021156. The 95% confidence interval for *σ*^2^ is (0.029047, 0.111978) indicating statistically significant unobserved heterogeneity, i.e., the existence of mortality deceleration. On the other hand, the MAP method estimates ${\hat{\theta }}_{MAP}={\left(0.006966,0.125440,0\right)}^{\prime }$, indicating the absence of unobserved heterogeneity. Comparing the goodness of fit of both methods speaks in favor of the MAP outcome: MAP’s MSE is by 37% lower than ML’s LSE (0.018691 for MAP vs 0.029958 for ML). It indicates that unobserved heterogeneity is negligible and that the gamma-Gompertz model is misspecified.

**Fig 3:**
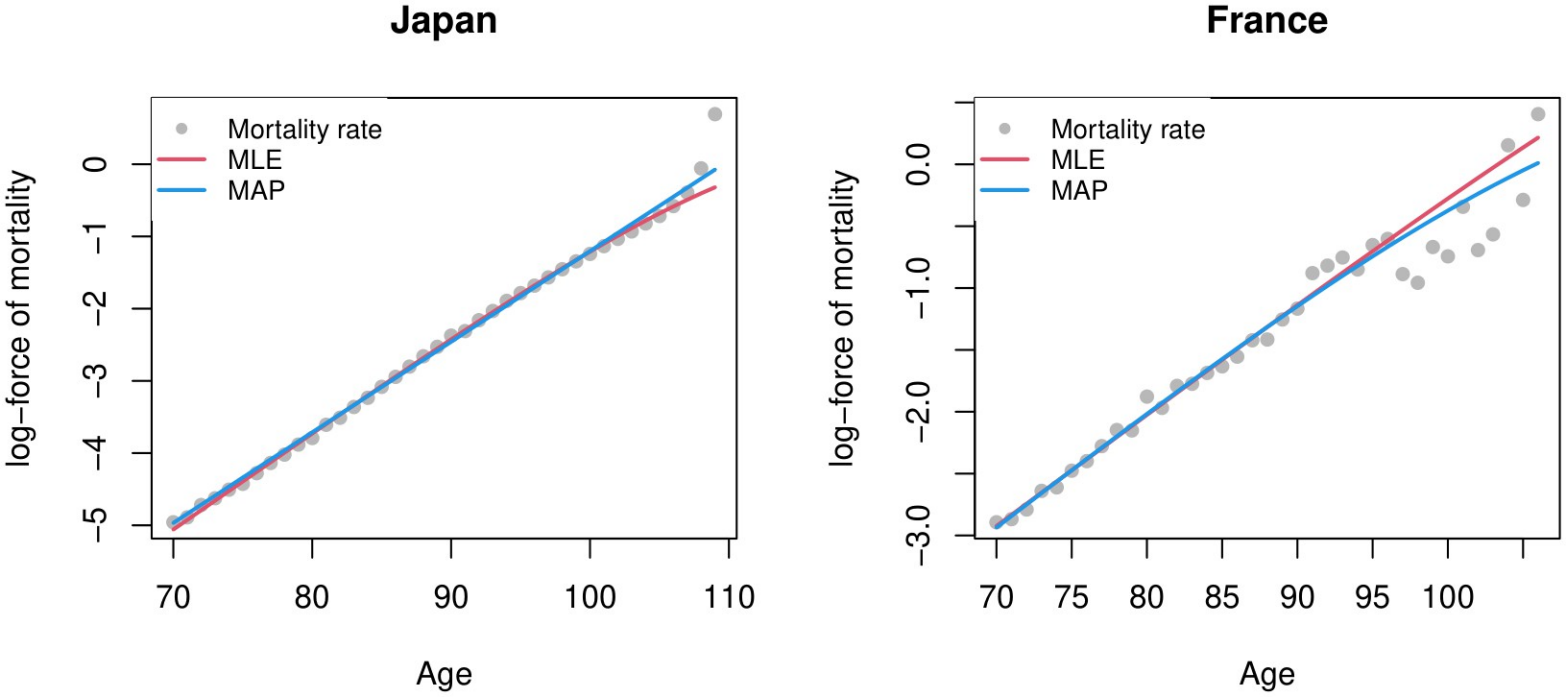
MAP and MLE estimates of the force of mortality for the Japanese population in 2009 and the Swedish population born in 1881, after age 70.

The left panel of Fig 3 shows that both methods estimate a similar logarithmic force of mortality at most ages. However, after age 100, the MLE deviates downward from the observed logarithmic death rates.

The MAP also provides a better fit and different conclusion for the cohort of French females born in 1848. While ML estimates ${\hat{\theta }}_{MLE}={\left(0.053748,0.090552,0.008604\right)}^{\prime }$ with *SE*(*a*) = 0.000317, *SE*(*b*) = 0.001273, *SE*(*σ*^2^) = 0.007562 and provides an MSE equal 0.046222, MAP estimates ${\hat{\theta }}_{MAP}={\left(0.053113,0.094921,0.036466\right)}^{\prime }$ and provides *MSE* = 0.034226, i.e., MAP’s MSE is by 26% smaller than ML’s MSE.

Furthermore, while the MAP estimate of *σ*^2^ suggests that there is non-negligible unobserved heterogeneity, the ML estimate and standard error for *σ*^2^ indicates the opposite: the amount of unobserved heterogeneity is not statistically significant. The right panel of Fig 3 shows the difference between these estimates. MAP’s estimate shows a leveling-off in the force of mortality, while the MLE shows a log-linear increase in the hazard function.

### 3.2 Comparison between MAP and ML estimators for different populations

To evaluate and compare empirically the performance of the ML and MAP methods we apply them to estimate the force of mortality for the male and female populations of France, Denmark, Sweden, Italy, Japan, Czechia, and the United States of America from 1950 to 2019, overall 980 populations. To access the goodness of fit we are using the MSE.

**Fig 4:**
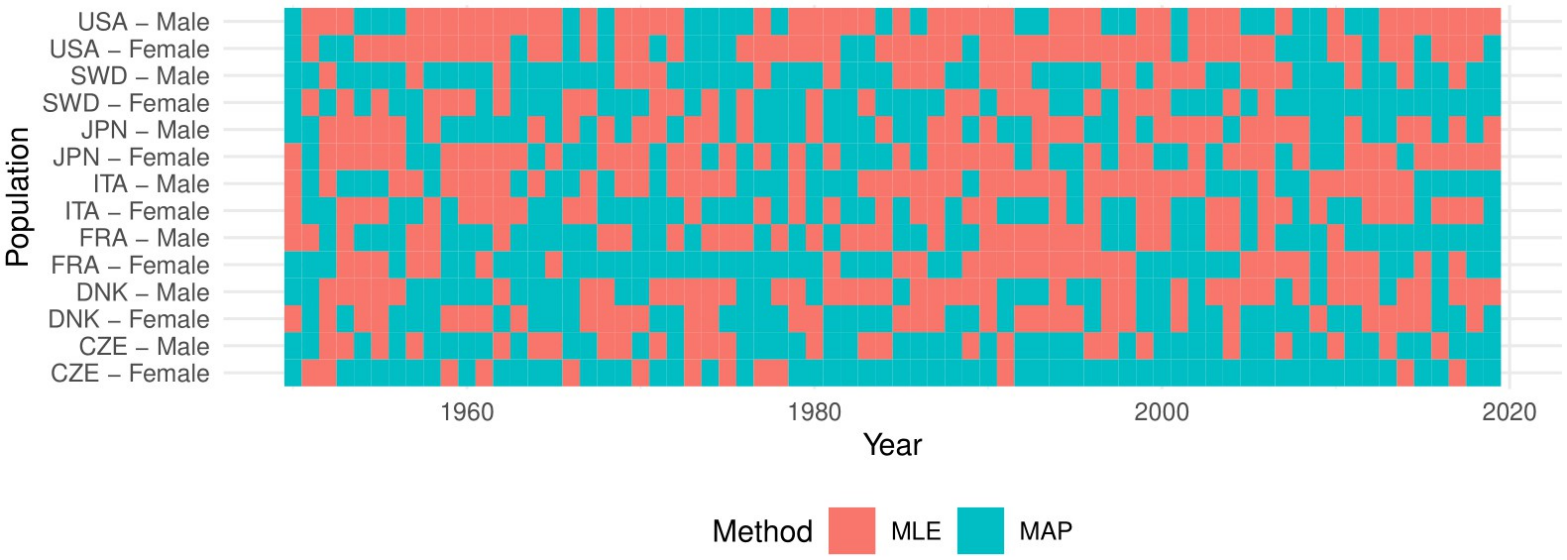
Method providing the best goodness of fit.

Fig 4 presents the method that provides a better fit (which has the smallest MSE). Overall both methods provide similar goodness of fit, with MAP providing a slightly lower MSE (on average 0.5% smaller than the ML method). Over the 980 populations, the MAP provides a better fit for 502 of them. For Czechia, Sweden, and France, the MAP provides a slightly better fit than the ML method. In general, within each country, the MAP-ML differences in MSE are small.

**Fig 5:**
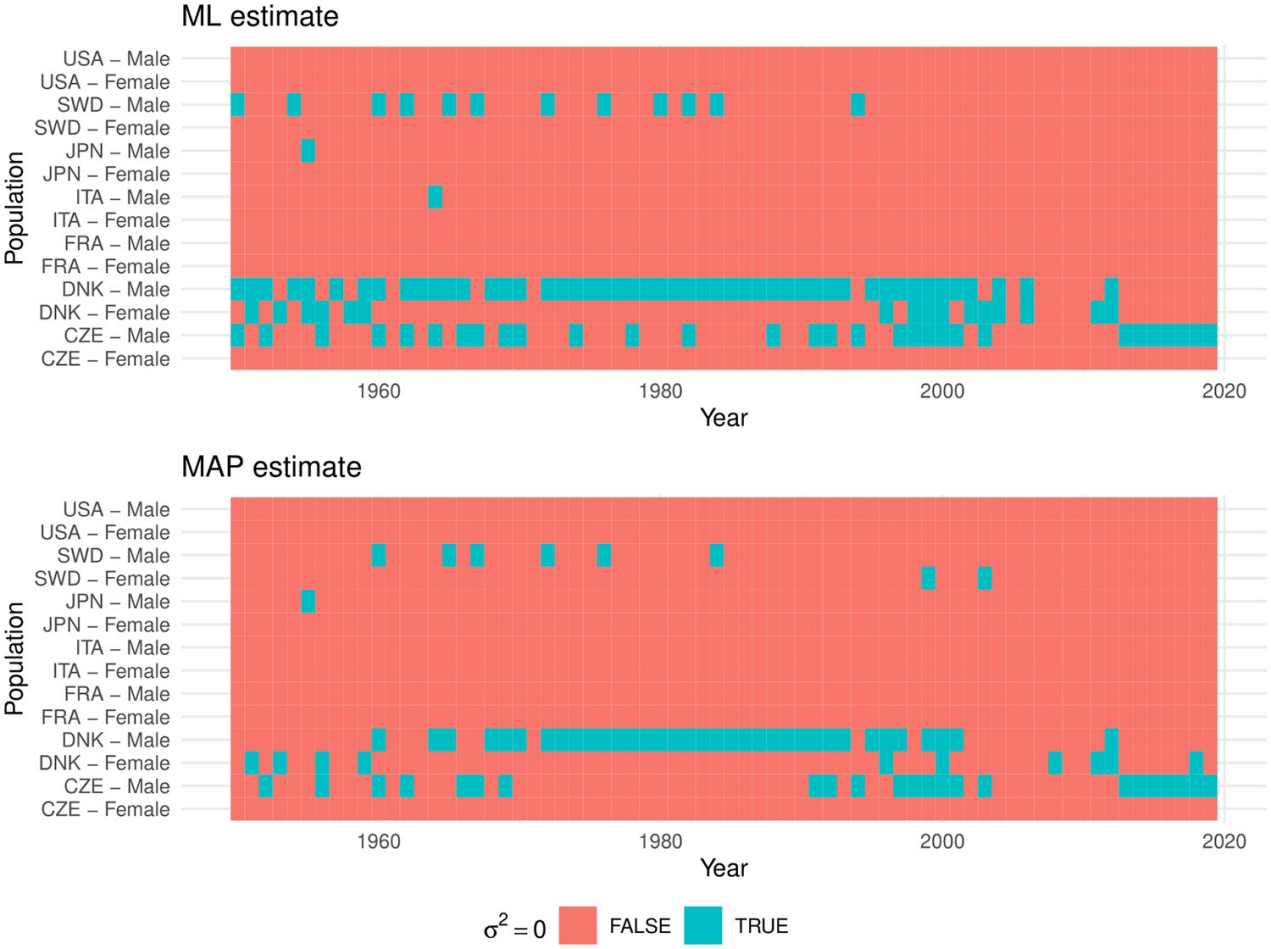
Statistical non-significance of *σ*^2^.

We also estimate the standard error for the ML estimates of *σ*^2^ to test if the parameter is not statistically significantly different from zero. We compare the results with the ones for the MAP estimate. Fig 5 presents for which populations *σ*^2^ is zero through the ML (top panel) and MAP method (bottom panel). From both methods, it is clear that the unobserved heterogeneity is statistically negligible only for some male populations—especially for Danes. This may result from the small number of males surviving to the oldest ages which leads to data fluctuations.

In about 96% of the populations, the methods agree on the statistical significance or non-significance of *σ*^2^. However, when the MAP estimates *σ*^2^ = 0, the ML provides positive confidence intervals, i.e., non-zero *σ*^2^-estimates, in 5.1948% of the cases, which corresponds to the chosen *α*-level (probability of Type I error) of 5% for those intervals.

## 4 Concluding remarks

Böhnstedt and Gampe introduced a formal procedure to identify whether *σ*^2^ > 0 or *σ*^2^ = 0 in a hypothesis testing setting: they studied the asymptotic properties of the maximum likelihood estimator and the likelihood ratio test (LRT) for *H*_0_: *σ*^2^ = 0 vs. *H*_1_: *σ*^2^ > 0 for the gamma-Gompertz model [25]. However, LRTs are based on the asymptotic distribution of the maximum likelihood estimator; hence its convergence depends on the sample size. Moreover, conclusions drawn from hypothesis tests are dependent on the arbitrary choice of the significance level or *p*-value.

We suggest an alternative method by considering the problem as model misspecification based on the Poisson likelihood function. We add a penalty function to the likelihood so that we make sure that ${\hat{\sigma }}^{2}$ is exactly 0 when there is no heterogeneity, and we present its Bayesian interpretation (MAP). We assume death counts to be Poisson-distributed [32], but alternative specifications might yield even higher accuracy. Examples of alternatives for the death-count distribution are the negative binomial and the one-parameter Bell distributions [47–49]. In these cases, the penalty parameter λ may not be equal to $\frac{1}{2}$. Instead, it requires careful adjustment, a process best carried out through a comprehensive simulation study as outlined by Li et al. [40], which ensures a robust calibration of the parameter for accurate results.

We take advantage of robust Monte Carlo simulations to measure the bias and standard deviation of the ML and MAP methods in scenarios with and without unobserved heterogeneity. We also compare the performance of both methods for estimating the gamma-Gompertz model parameters using actual mortality data from the Human Mortality Database. The two methods work almost equally well, the ML having a slight advantage, in the presence of unobserved heterogeneity. However, in the absence of the latter, the MAP method provides an estimate closer to 0 (${\hat{\sigma }}^{2}\approx 10^{-20}$) and a better fit to the model in comparison to ML. As a result, the method we propose here can be used as an alternative to likelihood ratio testing for the gamma-Gompertz model with *H*_0_: *σ*^2^ = 0 vs. *H*_1_: *σ*^2^ > 0. On the one hand, the MAP method does not depend on any asymptomatic distribution, its performance is not strongly affected by sample size, and it also does not depend on the arbitrary choice of the significance level. On the other hand, MAP provides similar estimates to the ones by ML when *σ*^2^ > 0 and more accurate estimates when *σ*^2^ = 0.

## Supporting information

S1 Appendix (PDF)
