# Supplementary material for: Using a penalized likelihood to detect mortality deceleration
Source: PLoS One. 2023 Nov 16;18(11):e0294428. doi: 10.1371/journal.pone.0294428 (PMC10653492; doi:10.1371/journal.pone.0294428)
Supplement: S1 Appendix — (PDF) [file pone.0294428.s001.pdf]

Appendices

Table 3. Simulation results: gamma-Gompertz model and sample size 2,000.

There is heterogeneity												
	MLE estimator						MAP estimator					
	Bias			Standard deviation			Bias			Standard deviation		
Parameter	a	b	σ^2	a	b	σ^2	a	b	σ^2	a	b	σ^2
(a_1, b_1, σ_1^2)	0.000089	-0.001172	-0.020361	0.000111	0.003286	0.047684	0.000104	-0.001709	-0.029261	0.000117	0.003465	0.051082
(a_1, b_1, σ_2^2)	0.000110	-0.002075	-0.048634	0.000128	0.003972	0.078621	0.000118	-0.002397	-0.055984	0.000129	0.003970	0.078467
(a_1, b_2, σ_1^2)	0.000104	-0.000906	-0.008883	0.000124	0.004826	0.047186	0.000120	-0.001603	-0.016820	0.000130	0.005032	0.049798
(a_1, b_2, σ_2^2)	0.000124	-0.001978	-0.029154	0.000139	0.005738	0.077069	0.000133	-0.002420	-0.036104	0.000140	0.005733	0.076901
(a_2, b_1, σ_1^2)	0.000030	-0.003810	-0.046694	0.000021	0.003024	0.045092	0.000034	-0.004407	-0.057147	0.000024	0.003388	0.052275
(a_2, b_1, σ_2^2)	0.000038	-0.005056	-0.097144	0.000023	0.003338	0.070146	0.000039	-0.005316	-0.103429	0.000024	0.003333	0.069973
(a_2, b_2, σ_1^2)	0.000027	-0.003998	-0.030152	0.000021	0.004259	0.043531	0.000030	-0.004673	-0.038167	0.000022	0.004481	0.046813
(a_2, b_2, σ_2^2)	0.000032	-0.005191	-0.065676	0.000025	0.005158	0.075375	0.000034	-0.005565	-0.071836	0.000025	0.005146	0.075141
There is no heterogeneity												
	MLE estimator						MAP estimator					
	Bias			Standard deviation			Bias			Standard deviation		
Parameter	a	b	σ^2	a	b	σ^2	a	b	$\sigma^2(10^{-12})$	a	b	$\sigma^2(10^{-12})$
(a_1, b_1, σ^2)	0.000015	-0.001225	0.000002	0.000013	0.001615	0.006546	0.000016	-0.001341	0.021800	0.000014	0.001693	0.000287
(a_1, b_2, σ^2)	0.000014	-0.001224	0.000002	0.000013	0.001833	0.007389	0.000016	-0.001312	0.018541	0.000014	0.001858	0.004010
(a_1, b_3, σ^2)	0.000014	-0.001323	0.000002	0.000013	0.001998	0.008857	0.000015	-0.001324	0.024195	0.000015	0.002098	0.001130
(a_2, b_1, σ^2)	0.000028	-0.000591	0.000003	0.000031	0.001703	0.009242	0.000031	-0.000983	0.000923	0.000033	0.001666	0.000003
(a_2, b_2, σ^2)	0.000029	-0.000837	0.000003	0.000031	0.001857	0.010440	0.000031	-0.000842	0.000731	0.000031	0.001804	0.000009
(a_2, b_3, σ^2)	0.000030	-0.000810	0.000004	0.000031	0.002067	0.011604	0.000035	-0.001196	0.000701	0.000034	0.002086	0.000008
(a_3, b_1, σ^2)	0.000034	-0.000398	0.000003	0.000046	0.001739	0.011618	0.000037	-0.000606	0.000220	0.000048	0.001681	0.000001
(a_3, b_2, σ^2)	0.000036	-0.000503	0.000005	0.000049	0.002062	0.013431	0.000040	-0.000670	0.000151	0.000048	0.001901	0.000002
(a_3, b_3, σ^2)	0.000040	-0.000238	0.000008	0.000050	0.002247	0.014168	0.000045	-0.000722	0.000108	0.000051	0.002061	0.000008

Table 4. Simulation results: gamma-Gompertz model and sample size 5,000.

There is heterogeneity												
	MLE estimator						MAP estimator					
	Bias			Standard deviation			Bias			Standard deviation		
Parameter	a	b	σ^2	a	b	σ^2	a	b	σ^2	a	b	σ^2
(a_1, b_1, σ_1^2)	0.000067	-0.000379	-0.004470	0.000071	0.002078	0.029046	0.000071	-0.000552	-0.007370	0.000072	0.002090	0.029281
(a_1, b_1, σ_2^2)	0.000070	-0.000582	-0.015178	0.000077	0.002506	0.049888	0.000074	-0.000708	-0.018078	0.000077	0.002504	0.049808
(a_1, b_2, σ_1^2)	0.000090	-0.000273	0.001254	0.000077	0.002969	0.028061	0.000095	-0.000509	-0.001483	0.000078	0.002982	0.028253
(a_1, b_2, σ_2^2)	0.000094	-0.000514	-0.007569	0.000083	0.003583	0.048230	0.000097	-0.000687	-0.010324	0.000084	0.003579	0.048152
(a_2, b_1, σ_1^2)	0.000014	-0.001320	-0.014568	0.000011	0.001786	0.026808	0.000014	-0.001465	-0.017208	0.000011	0.001795	0.027016
(a_2, b_1, σ_2^2)	0.000015	-0.001650	-0.033574	0.000013	0.002344	0.049087	0.000016	-0.001748	-0.036030	0.000013	0.002342	0.049016
(a_2, b_2, σ_1^2)	0.000014	-0.001299	-0.006177	0.000012	0.002706	0.026681	0.000015	-0.001505	-0.008713	0.000012	0.002717	0.026863
(a_2, b_2, σ_2^2)	0.000015	-0.001571	-0.019479	0.000014	0.003352	0.046735	0.000016	-0.001714	-0.021904	0.000014	0.003349	0.046666
There is no heterogeneity												
	MLE estimator						MAP estimator					
	Bias			Standard deviation			Bias			Standard deviation		
Parameter	a	b	σ^2	a	b	σ^2	a	b	$\sigma^2(10^{-12})$	a	b	$\sigma^2(10^{-12})$
(a_1, b_1, σ^2)	0.000008	-0.000312	0.000009	0.000008	0.001139	0.006564	0.000008	-0.000441	0.024410	0.000009	0.001142	0.000058
(a_1, b_2, σ^2)	0.000008	-0.000339	0.000011	0.000009	0.001290	0.007315	0.000009	-0.000497	0.024494	0.000009	0.001259	0.000179
(a_1, b_3, σ^2)	0.000008	-0.000237	0.000011	0.000009	0.001398	0.007896	0.000009	-0.000483	0.037635	0.000009	0.001417	0.000985
(a_2, b_1, σ^2)	0.000015	0.000055	0.000823	0.000021	0.001212	0.008106	0.000016	-0.000102	0.001137	0.000022	0.001184	0.000012
(a_2, b_2, σ^2)	0.000017	0.000028	0.000732	0.000022	0.001363	0.009211	0.000020	-0.000228	0.001954	0.000023	0.001319	0.001225
(a_2, b_3, σ^2)	0.000019	0.000122	0.000182	0.000022	0.001473	0.009371	0.000021	-0.000024	0.002634	0.000022	0.001387	0.000014
(a_3, b_1, σ^2)	0.000023	0.000212	0.001357	0.000033	0.001265	0.009088	0.000023	-0.000068	0.000266	0.000033	0.001200	0.000001
(a_3, b_2, σ^2)	0.000025	0.000198	0.000845	0.000034	0.001391	0.009982	0.000030	-0.000154	0.000558	0.000035	0.001332	0.000048
(a_3, b_3, σ^2)	0.000026	0.000127	0.000722	0.000035	0.001590	0.011029	0.000034	-0.000151	0.001303	0.000035	0.001464	0.003727
